# Update: cohort mortality study of workers highly exposed to polychlorinated biphenyls (PCBs) during the manufacture of electrical capacitors, 1940-1998

**DOI:** 10.1186/1476-069X-5-13

**Published:** 2006-05-22

**Authors:** Mary M Prince, Misty J Hein, Avima M Ruder, Martha A Waters, Patricia A Laber, Elizabeth A Whelan

**Affiliations:** 1 Centers for Disease Control and Prevention (CDC), National Institute for Occupational Safety and Health (NIOSH), Division of Surveillance, Hazard Evaluation and Field Studies, Industrywide Studies Branch, 4676 Columbia Parkway, Cincinnati, Ohio 45226, USA

## Abstract

**Background:**

The National Institute for Occupational Safety and Health previously reported mortality for a cohort of workers considered highly exposed to polychlorinated biphenyls (PCBs) between 1939 and 1977 at two electrical capacitor manufacturing plants. The current study updated vital status, examined liver and rectal cancer mortality previously reported in excess in this cohort and evaluated mortality from non-Hodgkin's lymphoma (NHL) and cancers of the stomach, intestine, breast, prostate, skin (melanoma) and brain reported to be in excess in other cohort and case-control studies of PCB-exposed persons.

**Methods:**

Mortality was updated through 1998 for 2572 workers. Age-, gender-, race- and calendar year-adjusted standardized mortality ratios (SMRs) and 95% confidence intervals (CI) were calculated using U.S., state and county referent rates. SMRs using U.S. referent rates are reported. Duration of employment was used as a surrogate for exposure.

**Results:**

Consistent with the previous follow-up, mortality from biliary passage, liver and gall bladder cancer was significantly elevated (11 deaths, SMR 2.11, CI 1.05 – 3.77), but mortality from rectal cancer was not (6 deaths, SMR 1.47, CI 0.54 – 3.21). Among women, mortality from intestinal cancer (24 deaths, SMR 1.89, CI 1.21 – 2.82) and from "other diseases of the nervous system and sense organs", which include Parkinson's disease and amyotrophic lateral sclerosis, (15 deaths, SMR 2.07, CI 1.16 – 3.42) were elevated. There were four ALS deaths, all women (SMR 4.35, CI 1.19–11.14). Mortality was elevated for myeloma (7 deaths, SMR 2.11, CI 0.84 – 4.34), particularly among workers employed 10 years or more (5 deaths, SMR 2.80, CI 0.91 – 6.54). No linear associations between mortality and duration of employment were observed for the cancers of interest.

**Conclusion:**

This update found that the earlier reported excess in this cohort for biliary, liver and gall bladder cancer persisted with longer follow-up. Excess mortality for intestinal cancer among women was elevated across categories of duration of employment; myeloma mortality was highest among those working 10 years or more. The small numbers of deaths from liver and intestinal cancers, myeloma and nervous system diseases coupled with the lack of an exposure-response relationship with duration of employment preclude drawing definitive conclusions regarding PCB exposure and these causes of death.

## Background

Polychlorinated biphenyls (PCBs) are synthetic chlorinated aromatic hydrocarbons produced commercially in the U.S. from 1929 to 1977 and used as electrical insulating and heat exchange fluids due to their high stability, dielectric properties, resistance to oxidation, and incombustibility [1,2]. Peak production of PCBs occurred in the early 1970s. PCBs were banned in 1977 from U.S. production and distribution because of concern about the persistence of PCBs in the environment and potential health risks [2]. PCBs remain a potential occupational exposure, especially to persons who repair electrical equipment.

The purpose of this study was to update the mortality experience of a cohort of electrical capacitor manufacturing workers considered highly exposed to PCBs and to re-examine the increased risks for mortality from cancers of the liver and rectum previously observed in this cohort, primarily among women employed in one plant [3,4]. These excesses had not been found to be related to duration of employment or time since first exposure in PCB-exposed jobs. Other *a priori *outcomes of interest for which previous studies indicated increased risks with respect to PCB exposure included mortality from all cancers [5], non-Hodgkin's lymphoma (NHL) [6], and breast [7], skin (melanoma) and brain cancer [8]. Recent studies have also associated PCB exposure with risk of prostate [9,10] and stomach and intestinal cancer [11].

## Methods

The study plants have been described previously [3,4]. Briefly, workers were employed for at least 90 days in electrical capacitor manufacturing at a New York plant (Plant 1) between January 1, 1946, and June 30, 1977, or at a Massachusetts plant (Plant 2) between January 1, 1939, and March 29, 1976)[3,4]. The study cohort for this analysis was restricted to 2572 workers (Plant 1: 973; Plant 2: 1599) who held jobs identified as having the highest, most direct exposure to PCBs (e.g., impregnation, sealing and testing capacitors) and excluded workers confirmed at the time of the original study to be solvent exposed [4]. PCBs at both plants included, at various times, Aroclor 1254 (54% chlorine), Aroclor 1242 (42% chlorine), and Aroclor 1016 (41% chlorine) [4], with less chlorinated varieties used more recently. At any given time, it is likely that more than one Aroclor was in use. At Plant 1, use of Aroclor 1254 was phased out in the 1950s and Aroclor 1016 was first used beginning in 1971[12]; at Plant 2, specific dates are not known. NIOSH conducted exposure surveys at both plants in the spring of 1977. At Plant 1 the time-weighted average (TWA) air samples ranged from 24 – 476 μg/m^3^. PCB levels were generally higher at Plant 2, with TWAs from 50 – 1260 μg/m^3 ^[4].

Vital status was updated through December 31, 1998. Vital status was ascertained by linking with records of the Social Security Administration, the Internal Revenue Service, the National Death Index (NDI) [13], the U.S. Postal Service and credit bureaus. Causes of death were obtained from NDI Plus and from death certificates. A qualified nosologist coded causes of death from death certificates according to the International Classification of Diseases (ICD) revision in effect at the time of death.

The mortality experience of the cohort was analyzed using the NIOSH personal computer life-table analysis system (PC-LTAS) [14,15]. Each cohort member accumulated person-years at risk (PYAR) beginning on January 1, 1940, or the date the worker achieved 90 days of potential PCB exposure, whichever was later. Person time ended at the date of death, the date the worker was lost to follow-up, or the end date of the study (December 31, 1998), whichever was earliest. Person time was first stratified into gender, race, 5-year age and 5-year calendar time strata and then multiplied by the appropriate U.S. death rates to generate expected numbers of deaths for each stratum. The resulting expected numbers of deaths were summed across strata to generate the expected number of deaths for each cause of death category and overall. NY county rates were created by combining three counties (Washington, Warren, and Saratoga), where 85% of Plant 1 workers resided (as of last known address), and MA county rates by combining two counties (Bristol and Plymouth), where 89% of Plant 2 workers resided. The mortality analysis was repeated using these county death rates, death rates from the state of New York (excluding New York City) for Plant 1 and death rates for Massachusetts for Plant 2. In addition to analyses using underlying cause of death, "multiple cause" of death (MCOD) rates were also used to examine non-cancer outcomes and cancers having high survival rates (e.g., prostate and breast cancers) [16]. For myeloma, NHL and melanoma, U.S. rate files begin on January 1, 1960, as do multiple cause, state and county rate files. For other causes of death, U.S. rate files begin on January 1, 1940.

The ratio of observed to expected number of deaths was expressed as the standardized mortality ratio (SMR). SMR 95% confidence intervals (CI) were computed assuming a Poisson distribution for the observed number of deaths. Statistical significance of the SMRs was computed using exact methods for 5 or fewer deaths. For greater numbers of observed deaths, the approximation method of Rothman and Boice [17] was used.

Duration of employment was used as a proxy for exposure to evaluate whether mortality was associated with length of exposure. Duration of employment was calculated, using re-coded work histories, by considering all time worked at the plant during the period of PCB use, not just time spent in jobs with the highest, most direct exposure to PCBs, as was done in the previous studies of this cohort [3,4]. The list of the job codes designated in the previous studies as having the highest and most direct PCB exposure was not available for this update.

Results are presented overall and stratified by gender, plant, duration of employment and time period of follow-up. Three duration of employment groups (<5, 5–9 and ≥ 10 years) were selected to allow comparisons with the previous update of this cohort [3]. For results stratified by time period of follow-up (original time period through 1982 and updated time period 1983 – 1998), we considered causes of death considered important by Brown and reported in his Table 3[3]. Trend tests were conducted for selected outcomes using methods specified in Breslow et al. [18], but only for those outcomes with at least 10 observed deaths.

There was some ambiguity as to the racial composition of the Plant 2 cohort, for which race was not coded in company records. In LTAS analyses, workers of "unknown" race are usually recoded 'white' for the analysis, and white-race rate files are used. However, the Plant 2 cohort, like the surrounding community, included many immigrants from the Cape Verde islands and the descendants of those immigrants [4]. Most Cape Verdeans are considered to be multiracial)[19] and therefore possibly 'non-white'. However, because many death certificates that listed the decedent's or decedent's parents' place of birth as Cape Verde listed race as "white" and because many cohort women, interviewed in 1997–2001 for an ancillary study, identified themselves as "white", we did not change the default LTAS recoding.

## Results

The previous update examined the mortality experience of 2588 workers (Plant 1: 981; Plant 2: 1607). Work histories were recoded for the present update. Sixteen workers (8 from each plant) were found to have worked fewer than 90 days and were excluded. Gender was corrected for 34 workers. The present update included 2572 workers. With 16 additional years of follow-up, 503 additional deaths (Plant 1: 194; Plant 2: 309) and 38,078 additional PYAR (Plant 1: 14,742; Plant 2: 23,336) were observed. Cohort demographics and employment characteristics are presented in Table 1. At Plant 1, more workers were male (61%). At Plant 2, the proportion of male workers increased over time from a minimum of around 10% in the early 1940s to about 37% in 1976. Because turnover was greater among men, men represent 41% of the total number of workers ever employed at Plant 2.

**Table 1:** Vital status and demographic characteristics of PCB-exposed workers stratified by gender and plant ^a ^

Characteristic	Combined plants						Plant 1		Plant 2	
					
	Total		Men		Women					
Number of workers	2572		1247	(48)	1325	(52)	973	(38)	1599	(62)
Race, n (%)										
White	2523	(98)	1239	(99)	1284	(97)	967	(99)	1556	(97)
Non-white	49	(2)	8	(1)	41	(3)	6	(1)	43	(3)

Vital status as of 12-31-1998, n (%)										
Deceased, cause of death known	787	(31)	348	(28)	439	(33)	305	(31)	482	(30)
Deceased, cause of death unknown	11	(<1)	7	(1)	4	(<1)	5	(1)	6	(<1)
Alive	1743	(68)	876	(70)	867	(65)	655	(67)	1088	(68)
Unknown	31	(1)	16	(1)	15	(1)	8	(1)	23	(1)

Age at death or date last observed (years)										
mean ± standard deviation	63.6 ± 13.0		59.8 ± 11.8		67.1 ± 13.2		64.1 ± 12.4		63.2 ± 13.4	
median (range)	64.1 (18.5 – 102.0)		58.6 (19.5 – 95.4)		69.0 (18.5 – 102.0)		64.8 (27.4 – 95.4)		63.8 (18.5 – 102.0)	

Time since last employment (years) ^b^										
median (range)	26.5 (0 – 52.6)		25.8 (0 – 51.8)		26.8 (0 – 52.6)		21.5 (0 – 52.6)		29.5 (0 – 49.0)	

Age at first employment (years)										
mean ± standard deviation	26.8 ± 10.2		25.1 ± 9.3		28.3 ± 10.7		29.0 ± 9.1		25.4 ± 10.5	
median (range)	23.3 (15.0 – 63.7)		21.8 (15.2 – 63.7)		25.9 (15.0 – 63.7)		26.4 (17.1 – 57.2)		20.5 (15.0 – 63.7)	

Duration of employment, n (%)										
90 days – <5 years	1261	(49)	674	(54)	587	(44)	338	(35)	923	(58)
5 years – <10 years	448	(17)	205	(16)	243	(18)	201	(21)	247	(15)
≥10 years	863	(34)	368	(30)	495	(37)	434	(45)	429	(27)

median (range)	5.2 (0.3 – 35.8)		3.9 (0.3 – 35.8)		6.1 (0.3 – 35.1)		8.7 (0.3 – 31.4)		3.5 (0.3 – 35.8)	

Time since first employment (years), n (%)										
< 10	55	(2)	36	(3)	19	(1)	22	(2)	33	(2)
10 – <15	44	(2)	20	(2)	24	(2)	14	(1)	30	(2)
15 – <20	64	(2)	34	(3)	30	(2)	28	(3)	36	(2)
≥20	2409	(94)	1157	(93)	1252	(94)	909	(93)	1500	(94)

median (range)	36.8 (0.6 – 60.0)		33.3 (0.6 – 59.5)		39.6 (1.7 – 60.0)		33.1 (0.6 – 53.0)		38.5 (0.6 – 60.0)	

Person-years at risk	93,623		42,826		50,797		33,834		59,789	

^a ^Number in parentheses indicates percent of total number of workers available for analysis.

^b ^Employment refers to time worked during the time period when PCBs were in use at the plants.

Women and men worked in different jobs, especially at Plant 1. At Plant 1, the top three job categories were: for male workers, plant engineering craftsman, tool and die maker, and treat room operator; for female workers, assemble capacitors, tester, and check worker. At Plant 2, the top three job categories were: for male workers, pre-assembly, post-assembly, and oven and kettle operator; for female workers, post-assembly, pre-assembly, and electrical work.

SMRs are presented for all workers combined and stratified by gender and plant in Table 2, for selected outcomes stratified by duration of employment in Table 3, and for selected outcomes stratified by time period of follow-up (original time period through 1982 and update time period 1983 – 1998) in Table 4. All tables present results based on U.S. mortality rates since results were similar using state and county rates, except as noted below. For 94% of the workers in this cohort, 20 years or more had elapsed since first employment; therefore, results were not stratified by time since first employment (TSFE) when examining trends with duration of employment.

**Table 2:** Observed deaths and SMRs for selected causes of death^a^, stratified by gender and plant, 1940–1998.^b^

Underlying cause of death (ICD-9)	Combined plants									Plant 1			Plant 2		
							
	Total			Men			Women								
	
	O	SMR	95% CI	O	SMR	95% CI	O	SMR	95% CI	O	SMR	95% CI	O	SMR	95% CI
All deaths	798	0.99	0.92 – 1.06	355	0.97	0.88 – 1.08	443	1.00	0.91 – 1.09	310	0.95	0.85 – 1.07	488	1.01	0.92 – 1.10
All cancers (140–208)	218	1.01	0.88 – 1.15	78	0.87	0.69 – 1.09	140	1.11	0.93 – 1.31	71	0.82	0.64 – 1.03	147	1.14	0.96 – 1.34
MN of buccal cavity and pharynx (140–149)	4	1.04	0.28 – 2.67	2	0.89	0.11 – 3.20	2	1.27	0.15 – 4.59	3	1.75	0.36 – 5.11	1	0.47	0.01 – 2.63
MN digestive system and peritoneum (150-159)	63	1.24	0.95 – 1.59	17	0.78	0.45 – 1.25	46	1.59	1.16 – 2.12	18	0.88	0.52 – 1.38	45	1.49	1.09 – 2.00
MN of esophagus (150)	2	0.55	0.07 – 2.00	0			2	1.67	0.20 – 6.03	0			2	1.03	0.12 – 3.72
MN of stomach (151)	5	0.84	0.27 – 1.97	4	1.35	0.37 – 3.45	1	0.34	0.01 – 1.87	0			5	1.46	0.47 – 3.40
MN of intestine, excluding rectum (152–153)	27	1.32	0.87 – 1.93	3	0.39	0.08 – 1.14	24	1.89	1.21 – 2.82	8	1.00	0.43 – 1.97	19	1.53	0.92 – 2.40
MN of rectum (154)	6	1.47	0.54 – 3.21	2	1.12	0.14 – 4.06	4	1.75	0.48 – 4.46	1	0.60	0.02 – 3.34	5	2.08	0.67 – 4.85
MN of biliary passages, liver and gall bladder (155–156)	11	2.11	1.05 – 3.77	4	1.86	0.51 – 4.77	7	2.28	0.92 – 4.69	4	1.92	0.52 – 4.92	7	2.23	0.90 – 4.59
MN of pancreas (157)	12	1.13	0.59 – 1.98	4	0.91	0.25 – 2.32	8	1.30	0.56 – 2.56	5	1.17	0.38 – 2.73	7	1.11	0.45 – 2.29
MN respiratory system (160–165)	60	1.03	0.79 – 1.33	30	0.90	0.61 – 1.28	30	1.21	0.82 – 1.73	25	0.95	0.62 – 1.41	35	1.10	0.76 – 1.53
MN of trachea, bronchus, and lung (162)	55	0.98	0.74 – 1.28	27	0.85	0.56 – 1.23	28	1.16	0.77 – 1.68	22	0.87	0.55 – 1.32	33	1.07	0.74 – 1.51
MN of breast (174–175)	15	0.59	0.33 – 0.98	0			15	0.60	0.33 – 0.98	2	0.27	0.03 – 0.97	13	0.73	0.39 – 1.25
MN of female genital organs (179–184)	21	1.29	0.80 – 1.97				21	1.29	0.80 – 1.97	5	1.07	0.35 – 2.50	16	1.38	0.79 – 2.24
MN of cervix uteri (180)	5	1.32	0.43 – 3.09				5	1.32	0.43 – 3.09	2	1.93	0.23 – 6.97	3	1.09	0.23 – 3.19
MN of other and unspecified parts of the uterus (179, 181, 182)	7	1.87	0.75 – 3.84				7	1.87	0.75 – 3.84	3	2.82	0.58 – 8.25	4	1.49	0.41 – 3.80
MN of ovary, fallopian tube, and broad ligament (183)	9	1.10	0.50 – 2.08				9	1.10	0.50 – 2.08	0			9	1.56	0.71 – 2.96
MN of prostate (185)	7	1.14	0.46 – 2.35	7	1.14	0.46 – 2.35				5	1.39	0.45 – 3.25	2	0.79	0.10 – 2.85
MN urinary organs (188–189)	12	1.50	0.77 – 2.62	8	1.80	0.77 – 3.54	4	1.12	0.31 – 2.88	4	1.13	0.31 – 2.88	8	1.79	0.77 – 3.54
MN of kidney (189.0–189.2)	8	1.82	0.79 – 3.60	5	2.12	0.69 – 4.96	3	1.48	0.30 – 4.32	2	1.05	0.13 – 3.80	6	2.42	0.88 – 5.26
MN of bladder and other urinary organs (188, 189.3–189.9)	4	1.10	0.30 – 2.82	3	1.43	0.29 – 4.18	1	0.66	0.02 – 3.64	2	1.21	0.15 – 4.37	2	1.01	0.12 – 3.65
MN of skin (172–173)	2	0.52	0.06 – 1.87	2	0.92	0.11 – 3.33	0			1	0.61	0.02 – 3.39	1	0.45	0.01 – 2.50
Melanoma (173)^c^	1	0.34	0.01 – 1.87	1	0.60	0.02 – 3.35	0			1	0.80	0.02 – 4.45	0		
MN of brain (191, 192)	3	0.50	0.10 – 1.47	2	0.70	0.08 – 2.53	1	0.32	0.01 – 1.80	2	0.82	0.10 – 2.96	1	0.28	0.01 – 1.58
MN of other and unspecified sites (194–199)	9	0.62	0.28 – 1.18	3	0.50	0.10 – 1.46	6	0.71	0.26 – 1.55	2	0.35	0.04 – 1.25	7	0.81	0.32 – 1.66
Neoplasms of lymphatic and hematopoietic tissue (200–208)	22	1.09	0.68 – 1.65	7	0.77	0.31 – 1.60	15	1.35	0.76 – 2.23	4	0.49	0.13 – 1.26	18	1.50	0.89 – 2.37
Leukemia and aleukemia (204–208)	4	0.53	0.14 – 1.36	2	0.57	0.07 – 2.07	2	0.50	0.06 – 1.79	0			4	0.90	0.24 – 2.29
Non-Hodgkin's lymphoma (200, 202)^c^	10	1.31	0.63 – 2.41	2	0.60	0.07 – 2.18	8	1.86	0.80 – 3.66	1	0.32	0.01 – 1.80	9	1.98	0.91 – 3.77
Myeloma (203)^c^	7	2.11	0.84 – 4.34	3	2.22	0.46 – 6.50	4	2.03	0.55 – 5.19	3	2.25	0.46 – 6.59	4	2.01	0.55 – 5.14
Diabetes mellitus (250)	16	0.88	0.50 – 1.43	4	0.63	0.17 – 1.61	12	1.02	0.53 – 1.78	5	0.73	0.24 – 1.72	11	0.97	0.48 – 1.74
Diseases of the nervous system and sense organs (320–389)	18	1.37	0.81 – 2.17	2	0.40	0.05 – 1.44	16	1.97	1.13 – 3.20	9	1.78	0.81 – 3.38	9	1.12	0.51 – 2.12
Other diseases of the nervous system and sense organs (320–337, 341–389)	16	1.35	0.77 – 2.19	1	0.22	0.01 – 1.21	15	2.07	1.16 – 3.42	8	1.75	0.75 – 3.44	8	1.10	0.47 – 2.17
Diseases of the heart (390–398, 402, 404, 410–414, 420–429)	286	1.06	0.94 – 1.19	150	1.17	0.99 – 1.38	136	0.96	0.81 – 1.14	120	1.08	0.89 – 1.29	166	1.05	0.90 – 1.22
Ischemic heart disease (410–414)	224	1.12	0.98 – 1.28	120	1.20	0.99 – 1.43	104	1.04	0.85 – 1.26	93	1.10	0.88 – 1.34	131	1.14	0.95 – 1.35
Hypertension with heart disease (402, 404)	4	0.46	0.13 – 1.18	1	0.33	0.01 – 1.85	3	0.53	0.11 – 1.55	1	0.32	0.01 – 1.77	3	0.54	0.11 – 1.59
Other diseases of the heart (420–423, 425–428, 429.2–429.9)	42	0.87	0.62 – 1.17	20	0.98	0.60 – 1.51	22	0.79	0.49 – 1.19	20	1.06	0.65 – 1.64	22	0.74	0.47 – 1.13
Other diseases of the circulatory system (401, 403, 405, 415–417, 430–459)	82	1.07	0.85 – 1.33	27	1.03	0.68 – 1.49	55	1.10	0.83 – 1.43	29	1.02	0.68 – 1.46	53	1.10	0.83 – 1.44
Diseases of the respiratory system (460–519)	46	0.79	0.58 – 1.05	19	0.76	0.46 – 1.19	27	0.81	0.53 – 1.18	21	0.88	0.54 – 1.34	25	0.73	0.47 – 1.07
Chronic and unspecified bronchitis (490, 491)	3	1.97	0.41 – 5.76	1	1.43	0.04 – 7.97	2	2.42	0.29 – 8.74	1	1.54	0.04 – 8.56	2	2.29	0.28 – 8.26
Emphysema (492)	8	1.15	0.50 – 2.28	4	1.10	0.30 – 2.81	4	1.22	0.33 – 3.11	5	1.59	0.51 – 3.71	3	0.79	0.16 – 2.32
Pneumoconiosis and other respiratory diseases (470–478, 494–519)	20	0.74	0.45 – 1.14	9	0.74	0.34 – 1.41	11	0.73	0.37 – 1.31	10	0.87	0.42 – 1.61	10	0.64	0.31 – 1.18
Diseases of the digestive system (520–579)	29	0.80	0.54 – 1.15	14	0.82	0.45 – 1.38	15	0.78	0.44 – 1.29	6	0.41	0.15 – 0.89	23	1.07	0.68 – 1.60
Cirrhosis of the liver (571)	17	1.02	0.59 – 1.63	10	1.06	0.51 – 1.95	7	0.96	0.39 – 1.99	4	0.56	0.15 – 1.42	13	1.37	0.73 – 2.34
Diseases of the genitourinary system (580–629)	13	1.04	0.55 – 1.78	5	1.15	0.37 – 2.69	8	0.98	0.42 – 1.93	2	0.43	0.05 – 1.56	11	1.39	0.69 – 2.49
Accidents (E800-E949)	31	0.75	0.51 – 1.06	19	0.67	0.40 – 1.05	12	0.91	0.47 – 1.60	18	1.07	0.63 – 1.69	13	0.53	0.28 – 0.91
Suicide (E950-E959)	20	1.37	0.83 – 2.11	14	1.36	0.74 – 2.28	6	1.39	0.51 – 3.02	12	1.90	0.98 – 3.32	8	0.96	0.41 – 1.90
Homicide (E960-E978)	4	0.74	0.20 – 1.90	4	1.00	0.27 – 2.56	0			2	0.95	0.11 – 3.42	2	0.61	0.07 – 2.21
Unknown cause of death	11			7			4			5			6		
Other	8	0.40	0.17 – 0.79	5	0.55	0.18 – 1.28	3	0.28	0.06 – 0.81	1	0.14	0.00 – 0.75	7	0.56	0.22 – 1.15

Abbreviations: ICD-9: *International Classification of Disease*s, Ninth Revision; O: observed number of deaths; SMR: standardized mortality ratio based on U.S. referent rates; CI: confidence interval; MN: malignant neoplasm.

^a ^Categories omitted from the table include tuberculosis (ICD-9 = 010–018, 2 deaths), benign and unspecified neoplasms (ICD-9 = 210–239, 2 deaths), diseases of the blood and blood forming organs (ICD-9 = 280–289, 4 deaths), mental, psychoneurotic, and personality disorders (ICD-9 = 290–319, 2 deaths), diseases of the skin and subcutaneous tissue (ICD-9 = 680–709, 1 death), musculoskeletal diseases (ICD-9 = 710–739, 1 death), and symptoms and ill-defined conditions (ICD-9 = 780–796, 798, 799, 4 deaths).

^b ^Person-years at risk and observed deaths among 2572 workers in jobs with potential for the highest, most direct exposure to PCBs began accruing in 1940.

^c ^Person-years at risk and observed deaths began accruing in 1960 for melanoma, non-Hodgkin's lymphoma and myeloma due to rate file restrictions.

**Table 3:** Observed deaths and SMRs for selected causes of death^a^, stratified by duration of employment^b^

Cause of death	Duration of employment									Trend p-value^c^
		
	< 5 years			5 – 9 years			≥10 years			
		
	O	SMR	95% CI	O	SMR	95% CI	O	SMR	95% CI	
All deaths	279	1.07	0.95 – 1.21	148	1.07	0.90 – 1.25	371	0.90	0.81 – 1.00	0.018
All cancers	73	1.08	0.84 – 1.35	41	1.06	0.76 – 1.44	104	0.95	0.78 – 1.15	0.38
MN of intestine, excluding rectum	7	1.16	0.47 – 2.39	6	1.69	0.62 – 3.67	14	1.30	0.71 – 2.17	0.97
Among women	7	1.94	0.78 – 4.00	6	2.46	0.90 – 5.36	11	1.66	0.83 – 2.97	0.61
MN of rectum	0			2	2.75	0.33 – 9.93	4	1.91	0.52 – 4.89	---
MN of biliary passages, liver and gall bladder	4	2.49	0.68 – 6.38	2	2.16	0.26 – 7.80	5	1.86	0.60 – 4.34	0.66
MN of breast	5	0.61	0.20 – 1.43	2	0.39	0.05 – 1.40	8	0.67	0.29 – 1.32	0.74
MN of prostate	1	0.64	0.02 – 3.55	0			6	1.58	0.58 – 3.44	---
Neoplasms of lymphatic and hematopoietic tissue	9	1.33	0.61 – 2.52	3	0.84	0.17 – 2.47	10	1.02	0.49 – 1.88	0.65
Non-Hodgkin's lymphoma^d^	4	1.63	0.44 – 4.16	3	2.32	0.48 – 6.78	3	0.78	0.16 – 2.27	0.25
Myeloma^d^	2	2.08	0.25 – 7.50	0			5	2.80	0.91 – 6.54	---
Other diseases of the nervous system and sense organs										
Underlying cause of death	1	0.27	0.01 – 1.48	3	1.56	0.32 – 4.56	12	1.95	1.01–3.40	0.039
Multiple cause of death^d^	6	0.51	0.19 – 1.10	4	0.65	0.18 – 1.66	28	1.37	0.91–1.98	0.011
Ischemic heart disease	69	1.22	0.95 – 1.54	47	1.44	1.06 – 1.91	108	0.97	0.80 – 1.18	0.057

Abbreviations: O: observed number of deaths; SMR: standardized mortality ratio; CI: confidence interval; MN: malignant neoplasm.

^a ^*A priori *causes of death with sufficient numbers to stratify plus significant new findings.

^b ^Person-years at risk and observed deaths among 2572 workers in jobs with potential for the highest, most direct exposure to PCBs began accruing in 1940.

^c ^Trend test performed for causes of death with 10 or more observed deaths.

^d ^Person-years at risk and observed deaths began accruing in 1960 for non-Hodgkin's lymphoma, myeloma, and multiple cause of death from other diseases of the nervous system and sense organs due to rate file restrictions.

**Table 4:** Observed number of deaths and SMRs for selected cancers, stratified by plant, gender and study periods.

Underlying cause of death	Combined plants									Plant 1			Plant 2		
							
	Total			Men			Women								
	
	O	SMR	95% CI	O	SMR	95% CI	O	SMR	95% CI	O	SMR	95% CI	O	SMR	95% CI
All cancers (1940 – 1982) ^a^	62	0.78	0.60 – 1.00	17	0.54	0.32 – 0.87	45	0.93	0.68 – 1.25	18	0.58	0.34 – 0.91	44	0.91	0.66 – 1.22
All cancers (1983 – 1998) ^b^	157	1.18	1.00 – 1.38	61	1.04	0.80 – 1.34	96	1.29	1.05 – 1.58	53	0.97	0.73 – 1.27	104	1.33	1.08 – 1.61
MN of stomach (1940 – 1982)	1	0.36	0.01 – 1.99	1	0.71	0.02 – 3.98	0			0			1	0.63	0.02 – 3.48
MN of stomach (1983 – 1998)	4	1.26	0.34 – 3.23	3	1.82	0.37 – 5.31	1	0.66	0.02 – 3.67	0			4	2.19	0.60 – 5.60
MN of intestine, excluding rectum (1940 – 1982)	8	1.04	0.45 – 2.05	1	0.38	0.01 – 2.14	7	1.37	0.55 – 2.83	3	1.03	0.21 – 3.02	5	1.04	0.34 – 2.43
MN of intestine, excluding rectum (1983 – 1998)	19	1.51	0.91 – 2.36	2	0.39	0.05 – 1.42	17	2.27	1.32 – 3.64	5	0.99	0.32 – 2.32	14	1.86	1.02 – 3.12
MN of rectum (1940 – 1982)	4	2.11	0.57 – 5.39	1	1.25	0.03 – 6.96	3	2.73	0.56 – 7.97	1	1.25	0.03 – 6.96	3	2.73	0.56 – 7.97
MN of rectum (1983 – 1998)	2	0.94	0.11 – 3.40	1	1.02	0.03 – 5.65	1	0.88	0.02 – 4.87	0			2	1.60	0.19 – 5.78
MN of biliary passages, liver and gall bladder (1940 – 1982)	5	2.63	0.85 – 6.14	1	1.43	0.04 – 7.96	4	3.33	0.91 – 8.53	1	1.43	0.04 – 7.96	4	3.33	0.91 – 8.53
MN of biliary passages, liver and gall bladder (1983 – 1998)	6	1.82	0.67 – 3.97	3	2.04	0.42 – 5.97	3	1.65	0.34 – 4.81	3	2.22	0.46 – 6.50	3	1.54	0.32 – 4.51
MN of pancreas (1940 – 1982)	2	0.54	0.07 – 1.95	1	0.63	0.02 – 3.48	1	0.48	0.01 – 2.65	1	0.67	0.02 – 3.71	1	0.45	0.01 – 2.53
MN of pancreas (1983 – 1998)	10	1.46	0.70 – 2.69	3	1.06	0.22 – 3.11	7	1.74	0.70 – 3.58	4	1.45	0.39 – 3.71	6	1.47	0.54 – 3.19
MN of respiratory system (1940 – 1982)	10	0.59	0.28 – 1.09	5	0.45	0.15 – 1.05	5	0.86	0.28 – 2.01	7	0.88	0.35 – 1.80	3	0.34	0.07 – 0.99
MN of respiratory system (1983 – 1998)	50	1.23	0.92 – 1.63	25	1.12	0.72 – 1.65	25	1.38	0.89 – 2.04	18	1.01	0.60 – 1.59	32	1.41	0.97 – 1.99
MN of urinary organs (1940 – 1982)	4	1.43	0.39 – 3.66	4	2.50	0.68 – 6.40	0			2	1.67	0.20 – 6.02	2	1.25	0.15 – 4.52
MN of urinary organs (1983 – 1998)	8	1.52	0.66 – 3.00	4	1.35	0.37 – 3.46	4	1.74	0.48 – 4.46	2	0.86	0.10 – 3.11	6	2.05	0.75 – 4.45
Neoplasms of lymphatic and hematopoietic tissues (1940 – 1982)	5	0.68	0.22 – 1.58	1	0.29	0.01 – 1.64	4	1.00	0.27 – 2.56	0			5	1.11	0.36 – 2.59
Neoplasms of lymphatic and hematopoietic tissues (1983 – 1998)	17	1.38	0.80 – 2.20	6	1.08	0.40 – 2.36	11	1.61	0.80 – 2.88	4	0.79	0.22 – 2.02	13	1.78	0.95 – 3.05
MN of breast (1940 – 1982)	9	0.77	0.35 – 1.46	0			9	0.77	0.35 – 1.46	1	0.30	0.01 – 1.69	8	0.96	0.42 – 1.90
MN of breast (1983 – 1998)	6	0.46	0.17 – 1.01	0			6	0.46	0.17 – 1.01	1	0.25	0.01 – 1.41	5	0.55	0.18 – 1.29
MN of female genital organs (1940 – 1982)	7	0.85	0.34 – 1.76				7	0.85	0.34 – 1.76	1	0.43	0.01 – 2.42	6	1.02	0.37 – 2.21
MN of female genital organs (1983 – 1998)	14	1.85	1.01 – 3.11				14	1.85	1.01 – 3.11	4	1.74	0.47 – 4.44	10	1.91	0.91 – 3.50

Abbreviations: O: observed number of deaths; SMR: standardized mortality ratio based on U.S. referent rates; CI: confidence interval; MN: malignant neoplasm.

^a ^Results for vital status follow-up from 1940 through 1982 based on 2588 workers in jobs with the potential for the highest, most direct exposure to PCBs and computed from observed and expected deaths reported in Table 3 of Brown (1987).

^b ^Results for vital status follow-up from 1983 through 1998 based on 2257 PCB exposed workers (current cohort of 2572 workers [2588 workers less 16 with < 90 days of employment based on the recoded work histories] in jobs with the potential for the highest, most direct exposure to PCBs less 291 workers who died and 24 workers lost to follow-up prior to 1983).

### *A priori *causes of interest

Overall mortality was similar to expected (798 deaths, SMR 0.99, CI 0.92 – 1.06) as was all cancer mortality (218 deaths, SMR 1.01, CI 0.88 – 1.15); however, cancer mortality was significantly elevated in the update time period (157 deaths, SMR 1.18, CI 1.00 – 1.38). Cancers of the biliary passages, liver and gall bladder (henceforth referred to as liver cancer) were significantly elevated in the cohort (11 deaths, SMR 2.11, CI 1.05 – 3.77). Elevations in liver cancer were observed for both genders and both plants. The liver cancer elevation persisted across duration of employment categories but did not increase with duration of employment. Six new liver cancers were observed in the updated time period. Mortality from rectal cancer remained elevated in the cohort (6 deaths, SMR 1.47, CI 0.54 – 3.21), although only 2 new rectal cancers were observed in the updated time period and the elevation was somewhat reduced from the original study. The excess was largely due to an excess of rectal cancer deaths among women at Plant 2 in the original follow-up period. Mortality from NHL was moderately elevated in the cohort (10 deaths, SMR 1.31, CI 0.63 – 2.41), especially among women at Plant 2, but the highest NHL mortality was observed among workers with less than 10 years of employment.

Results for other *a priori *outcomes were not remarkable. Breast cancer was reduced in the cohort overall (15 deaths, SMR 0.59, CI 0.33 – 0.98) and did not increase with duration of employment. Two deaths from skin cancer (one malignant melanoma) and three deaths from brain cancer were observed in the cohort.

### Other cancer mortality

Prostate cancer was not elevated in the cohort (Table 2), but a modest elevation was observed among workers with 10 years or more of employment (6 deaths, SMR 1.58, CI 0.58 – 3.44). Mortality from cancer of the intestine was elevated, but only among women (24 deaths, SMR 1.89, CI 1.21 – 2.82). The intestinal cancer elevation among women persisted in each duration of employment category but did not increase with duration of employment. The excess was primarily due to intestinal cancer deaths among women at Plant 2 in the updated time period. Other cancer outcomes to note include myeloma and cancers of the kidney and female genital organs. Mortality from myeloma was somewhat elevated (7 deaths, SMR 2.11, CI 0.84 – 4.34), and, although there were too few deaths to test for a trend in mortality, the highest elevation was among workers employed for 10 years or more. Mortality from kidney cancer was somewhat elevated (8 deaths, SMR 1.82, CI 0.79 – 3.60). Mortality from cancers of the female genital organs was elevated in the time period since the last update (14 deaths, SMR 1.85, CI 1.01–3.11).

### Other causes of death

Other disease outcomes to note include diseases of the nervous system and sense organs, ischemic heart disease, and diseases of the digestive system. Mortality from other diseases of the nervous system and sense organs, a cause of death category that includes Parkinson's disease and amyotrophic lateral sclerosis, was elevated, particularly among women (15 deaths, SMR 2.07, CI 1.16 – 3.42). There were four ALS deaths, all women (SMR 4.35, CI 1.19–11.14). In the multiple cause of death analysis, which considered all causes listed on the death certificate, an additional 23 deaths were observed for other diseases of the nervous system and sense organs and an increasing trend was observed with duration of employment. A more detailed analysis can be found in Steenland et al[20] Mortality from ischemic heart disease was elevated, particularly among men (120 deaths, SMR 1.20, CI 0.99 – 1.43); however, the elevation was limited to workers with less than 10 years of employment. Mortality from diseases of the digestive system was reduced, particularly among Plant 1 workers (6 deaths, SMR 0.41, CI 0.15 – 0.89).

Mortality from accidents, primarily transportation accidents, was elevated among Plant 1 workers, especially when state rates were used (12 deaths, SMR 2.01, CI 1.04 – 3.51); however, mortality from accidents was reduced among Plant 2 workers (13 deaths, SMR 0.53, CI 0.28 – 0.91). Mortality from suicide was elevated among Plant 1 workers, with the elevation even more pronounced when state rates were used (11 deaths, SMR 2.79, CI 1.39–4.99).

## Discussion

An additional 16 years of mortality experience has been examined in this study, totalling 59 years of observation time since 1940. The main goal of the study was to examine mortality from liver and rectal cancers previously found to be in excess among workers exposed to PCBs. Mortality patterns within this cohort corroborated other studies showing excesses of liver cancer [21] and intestinal cancer [5,11] among PCB-exposed workers, but this study did not show that these excesses were related to duration of employment. In contrast to other studies of PCB exposure, only moderate excesses were found for NHL [6,22,23] and prostate cancer [9,10], and none for cancers of the skin [8,24], brain [8,25], or breast [26].

Results cannot confirm whether observed excesses in mortality for liver and intestinal cancers, myeloma, and diseases of the nervous system are related to PCB exposure due to limited sample sizes, lack of exposure-response with duration of employment, and lack of information on potential confounders. Although data on smoking and alcohol use are not available for this cohort, there is some suggestion that these factors may not significantly contribute to the observed excesses of liver and intestinal cancers or myeloma. Mortality from some diseases associated with smoking and alcohol (cirrhosis of liver and respiratory diseases) was not elevated, although heart disease mortality, particularly ischemic heart disease in men, did not show a "healthy worker" effect [27]. Myeloma risk has not been associated with smoking and alcohol use [28,29], but it has been linked to occupational chemical exposures [30,31]. It is also unlikely that regional differences contributed to the observed excesses in mortality from intestinal and liver cancers or myeloma since state- and county-based SMRs (not shown) were similar to those using U.S. referent rates.

We cannot rule out the possibility that other factors, including ethnic differences, may have contributed to the observed excesses of intestinal and liver cancers and myeloma [32]. The ethnic make-up of the Plant 2 workforce reflected that of the general population in the area, which was largely Cape Verdean and Portuguese [4]. The Cape Verde population was reported to be 71% Creole (mulatto), 28% African, and 1% European [19]. Cape Verde may share with other countries of West Africa a substantial risk of liver cancer, due in large part to hepatitis infection [33]. First-generation emigrants from West Africa retain an increased risk of liver cancer [34]. If most of the Cape Verdean workers at Plant 2 were born in the United States, their baseline rates of liver cancer (absent exposure) would be similar to those in the U.S., as liver and intestinal cancer rates in the offspring of immigrants are closer to those in their parents' adopted country than in their ancestral country [35]. It is unlikely that ethnic differences account for our results because additional liver cancer deaths have occurred among Plant 1 workers and males during the update period, suggesting that any initial differences in risk by plant and gender may have diminished over time with increased follow-up and latency (Table 4). Exposure to PCBs appears to have been higher in Plant 2 than Plant 1 [3,4] and both plants used the same Aroclor formulations. If PCBs have a causal relationship with liver cancer one would expect more cancer in a more highly exposed population.

In the current update, new findings include observed excesses of myeloma and diseases of the nervous system among women, neither of which has been reported in other studies of PCB-exposed workers. Associations have been reported between PCB exposure and Parkinson's or other neuropathology [36,37] and between dioxin, structurally similar to some PCB congeners, and myeloma [38]. Although based on small numbers of deaths, SMRs for myeloma were consistently elevated among men and women at both plants. The excess in mortality from diseases of the nervous system requires further exploration in other PCB-exposed cohorts, in light of the fourfold excess in ALS mortality as well as excess Parkinson's and dementia deaths in this highly exposed cohort. Our separate mortality analysis of neurodegenerative disease mortality among a large (n = 17,247) cohort of PCB-exposed workers which included all the workers in this study as well as other workers at these plants and workers at a third capacitor manufacturing plant found additional excess deaths among the workers with relatively higher cumulative exposure [20].

This study cannot fully evaluate any association between PCB exposure and breast cancer, a subject of ongoing interest in the environmental exposure literature [39,40], absent data on generally acknowledged breast cancer risk factors, such as parity and ages at menarche, first birth, and menopause. We have initiated a study of breast cancer among women occupationally exposed to PCBs with an interview component to obtain information on known risk factors.

Study limitations include the small cohort size and low number of deaths, lack of data on potential confounders and possible misclassification of exposure due to use of duration of employment as a surrogate for cumulative PCB exposure. Internal analyses using an internal referent group were not performed, since the cohort, by definition, included only workers who were highly exposed. Given these limitations, we will explore exposure-response relationships in an expanded cohort of workers at these plants with a wider range of potential PCB exposures. These analyses, which will be reported elsewhere, will use newly created job exposure matrices developed to estimate cumulative exposure to PCBs for workers at the two plants.

## Conclusion

Our results are consistent with previous studies of this cohort which also reported significantly elevated mortality from liver cancers, which was not found to be associated with duration of employment. In this update, the SMR for rectal cancer was elevated but lower than that observed in the last update and was not related to duration of employment. Increased mortality was also observed for myeloma and intestinal cancer (among women), but these elevations were not associated with duration of employment in a linear fashion. Mortality from diseases of the nervous system was associated with duration of employment. NHL and prostate cancer mortality were only moderately elevated. There was no evidence of increased mortality risk from cancers of the breast, skin or brain due to PCB exposure in this cohort.

## Authors' contributions

MMP wrote the draft manuscript and worked on manuscript revisions, MJH performed the statistical analyses and worked on manuscript revisions, AMR, MAW, and EAW contributed to the draft manuscript and worked on manuscript revisions, PAL built data structures, implemented follow-up procedures, and prepared the data for analysis. All authors reviewed the final manuscript.

## Disclaimer

The findings and conclusions in this report are those of the authors and do not necessarily represent the views of the National Institute for Occupational Safety and Health.

## Competing interests

The author(s) declare that they have no competing interests.
